# Administration of microencapsulated *Enterococcus faecium* ABRIINW.N7 with fructo-oligosaccharides and fenugreek on the mortality of tilapia challenged with *Streptococcus agalactiae*

**DOI:** 10.3389/fvets.2022.938380

**Published:** 2022-08-01

**Authors:** Yousef Nami, Mahdi Kahieshesfandiari, Gilda Lornezhad, Amir Kiani, Daniel Elieh-Ali-Komi, Mahdieh Jafari, Mehdi Jaymand, Babak Haghshenas

**Affiliations:** ^1^Department of Food Biotechnology, Branch for Northwest and West Region, Agricultural Biotechnology Research Institute of Iran, Agricultural Research, Education and Extension Organization (AREEO), Tabriz, Iran; ^2^Department of Aquaculture, Faculty of Agriculture, University Putra Malaysia, Selangor, Malaysia; ^3^Department of Medicine, School of Medicine, Kermanshah University of Medical Sciences, Kermanshah, Iran; ^4^Regenerative Medicine Research Center (RMRC), Kermanshah University of Medical Sciences, Kermanshah, Iran; ^5^Department of Animal, Marine and Aquatic Biology and Biotechnology, Faculty of Life Sciences and Biotechnology, Shahid Beheshti University, Tehran, Iran; ^6^Nano Drug Delivery Research Center, Kermanshah University of Medical Sciences, Kermanshah, Iran

**Keywords:** *Streptococcus agalactiae*, microencapsulated *E. faecium*, fenugreek, tilapia, fructooligosaccharide, aquaculture

## Abstract

We investigated the probiotic potential of a microencapsulated *Enterococcus faecium* ABRIINW.N7 for control of *Streptococcus agalactiae* infection in hybrid (*Oreochromis niloticus* × *Oreochromis mossambicus*) red tilapia. A two-phase experiment approach was completed in which *E. faecium* bacteria were propagated, from which a culture was isolated, identified using molecular techniques, and microencapsulated to produce a stable commercial fructooligosaccharide (FOS) and fenugreek (Fk) product of optimal concentration. The FOS and Fk products were assessed in a 90-days *in vivo* challenge study, in which red hybrid tilapia were allocated to one of five treatments: (1) No *Streptococcus agalactiae* (Sa) challenge (CON); (2) Sa challenge only (CON^+^); (3) Sa challenge in a free cell (Free Cell); (4) Sa challenge with 0.8% (w/v) Alginate; (5) Microencapsulated FOS and Fk. *In vitro* results showed high encapsulation efficiency (≥98.6 ± 0.7%) and acceptable viability of probiotic bacteria within the simulated fish digestive system and high stability of viable cells in all gel formulations (34 < SR% <63). *In vivo* challenges demonstrated that the FOS and Fk products could be used to control *S. agalactiae* infection in tilapia fish and represented a novel investigation using microencapsulation *E. faecium* as a probiotic diet for tilapia fish to control *S. agalactiae* infection and to lower fish mortality. It is recommended that local herbal gums such as 0.2% Persian gum and 0.4% Fk in combination with 0.8% alginate (Formulation 7) can be used as a suitable scaffold and an ideal matrix for the encapsulation of probiotics. These herbal gums as prebiotics are capable of promoting the growth of probiotic cells in the food environment and digestive tract.

## Introduction

Tilapia fish has become one of the most favored species for generating human food due to providing protein, critical vitamins, and minerals. Furthermore, tilapia is commonly utilized as a study model due to its short reproductive cycle, quick development, and strong tolerance to most common illnesses, stress, temperature change, and water quality (1). Continuous selective breeding produces hybrid red tilapia from chosen tilapia species of the genus *Oreochromis* (*Oreochromis niloticus, Oreochromis mossambicus*) (2).

Feeding and disease management are two of the most difficult aspects of tilapia aquaculture (3). Protozoa, metazoans, crustaceans, bacterial, fungal, and viral diseases, as well as high mortality rates, imperil the tilapia aquaculture business and result in significant financial loss (4).

Streptococcal bacteria are a well-known pathogen of facultative anaerobes in both produced and wild tilapia populations. *Streptococcus iniae, Streptococcus shiloi*, and *Streptococcus agalactiae* are bacteria from the Streptococcaceae family that are widely found in hot climes and are a major cause of septicemia and meningoencephalitis in many fish species, including tilapia (5). *Streptococcus agalactiae* can cause 50−75%, and in some cases up to 100%, deaths in a variety of fish (6).

Meningitis is the most common clinical symptom in tilapia infected with *S. agalactiae*. It is characterized by neurological disorders such as constant and abnormal swimming, exophthalmia and corneal opacity, and pathological changes in meningeal congestion and granulocyte infiltration, as well as pathological changes in meningeal congestion and granulocyte infiltration.

Vaccination, antibiotic treatment, antimicrobial compounds, dietary supplements, probiotics, prebiotics, nonspecific immunostimulants, medicinal plant products, genetically resistant stock and transgenic fish, water disinfection, biological control, and animal movement control are the main prevention and control strategies for marine infectious diseases.

Oral probiotic supplementation to lessen antimicrobial resistance is one of the most effective approaches to reduce the prevalence of major infectious illnesses in tilapia (7). To be commercialized, probiotic cells must be resistant to the enzymatic conditions of the fish digestive system and have appropriate stability under a variety of temperature and humidity settings. These objectives can be fulfilled by microencapsulating probiotics and employing suitable protective techniques and matrixing materials to enable controlled release.

Microencapsulation defines as packaging of materials and particles with the protective coating membranes or shells. It resulted to increase the stability of materials and particles, specific and controlled realization, masking the undesirable odors and inhibition of oxidative activity (8). Probiotics have long been thought to be beneficial to aquatic species' development, health, and survival. Skin, gills, mucus of aquatic animals, surroundings or commercial items, and culture collections are the major sources of suitable probiotics in aquaculture (9). Probiotics have several advantages in aquaculture, including (a) altering the surrounding microbial population or host-associated microbial communities, (b) increasing nutritional value, and (c) improving the immune response of aquatic animals against illnesses (10).

Enterococci are common bacteria found in the gastrointestinal tracts of humans and animals, and have been used as probiotics in the food business (11). When choosing helpful bacteria, Enterococci demonstrated extraordinary bacteriocins activity, which has been recommended as a novel probiotic feature (12). *Enterococcus faecium* is a Gram-positive lactic acid bacteria belonging to the genus *Enterococcus*. Generally, *E. faecium* is a ubiquitous commensal microorganism commonly found in the gut of animals and humans. *Enterococcus faecium* has long been recognized for its probiotic benefits and is widely used around the world. One of the major benefits of *E. faecium* is that it is uniquely suited to survive the digestive process and flourish in the gut. Probiotics have the most positive benefits when they survive the gastrointestinal system and stay functional at their target location (13).

Conditions such as extreme heat, excessive humidity, and dryness, on the other hand, may reduce the positive benefits of probiotics. By ion exchange with a negatively charged alginate (ALG) structure, herbal-based hydrogels such as Persian Gum (PG) may create microencapsulated beads with exceptional endurance (14). PG is a polysaccharide comprised of galactose, (13)-D–Galp, and rhamnose that is released by mountain almond (*Amygdalus scoparia Spach*; synonymous: *Prunus scoparia Spach*) trees that forms a hydrogel in normal physiological conditions. PG has long been used to alleviate joint swelling, toothaches, and coughs, and it might be considered a candidate for microencapsulation if correctly adapted to poly (AAm) utilizing N,N′-Methylenebisacrylamide (MBAA), and ammonium persulfate (APS) (9).

The most often used coating material for probiotic encapsulation is ALG. Alginate is a liner biopolymer which extracted from the algae. This natural gel is low cost, non-toxic, thermo stable, biocompatible and approved as the safe food additive. ALG, on the other hand, has drawbacks such as high porosity and sensitivity to low acidic conditions (10, 11). Alginate gel is susceptible to the verse chemical conditions at fermentation time then by adding the poly cationic herbal gels, its releasing rate and stability can be improved. To overcome these weaknesses of alginate, the combination of alginate with other natural hydrogels such as PG (15) and fenugreek (16) has been used to encapsulate probiotic bacteria.

The fenugreek polymer (biopolymer extracted from fenugreek seeds), is composed of D-mannan (backbone) and d-galactopyranosyl groups (side chains). This herbal gum shows the health promoting effects and prescribed for treatment of diabetes and regulation of cholesterol level. The prebiotic properties of fenugreek gum have led to its use in animal husbandry and nutritional supplements (17). Furthermore, because of its stability and emulsifying activity (18), this gel is appropriate for use in the food sector and in encapsulation processes (19).

The overall purpose of this study was to assess probiotic potential qualities and half-life estimation of microencapsulated *E. faecium* against *S. agalactiae* in challenged red hybrid tilapia fish using ALG-PG microcapsules. Furthermore, under tilapia digestion circumstances and throughout storage time, the morphological properties, encapsulation effectiveness, anti-pathogenicity, and probiotic cell survival were studied. This is the first study to employ a microencapsulated *E. faecium* probiotic-supplemented meal to reduce the mortality rate of *S. agalactiae*-infected tilapia fish.

## Materials and methods

All protocols and animal experiments in this study were approved by Kermanshah University of Medical Sciences' Committee on the Ethics of Animal Experiments (Approval ID: IR.KUMS.REC.1399.531), and were carried out in accordance with Kermanshah University of Medical Sciences' guidelines and regulations (https://ethics.research.ac.ir/docs/pages/Guideline-Res.pdf) and the ARRIVE guidelines for the involvement of animals (fish). This was a two-part investigation, with the first phase consisting of an *in-vitro* research in which bacteria were cultivated and an isolate was generated and identified using molecular methods. Using an *in vitro* assay to manufacture commercial fructooligosaccharides (FOS) and Fk by standard microencapsulation, this isolate was utilized to create a stabilized probiotic that was incorporated at an appropriate dose. In a 90-days *in vivo* feeding trial, the effectiveness of this was determined (phase 2).

### Phase 1: *In vitro* assay

#### Media and materials

University Putra Malaysia (UPM) provided the *S. agalactiae* (ATCC 13813). Fk and PG were purchased locally (Iran). Sodium hydrogen phosphate, hydrochloric acid, calcium chloride, sodium hydroxide, de Man Rogosa Sharpe (MRS) broth, MRS agar, and Acrylamide (AAm) (Merck, Germany) and N, N-methylene bis-acrylamide (NN-MBAAm), ammonium persulphate (APS), fructooligosaccharides (FOS), oxgall, and sodium ALG were purchased (Sigma-Aldrich, Germany).

#### Molecular identification and strain characterization of *E. faecium*

The *E. faecium* ABRIINW.N7 strain was isolated from ewe colostrum, cultured, and amplified on MRS broth medium for 48 h at 37°C. The genomic DNA was extracted based on the methodology described by Henning et al. (12). The primers (F 5′-AGAGTTTGATCCTGGCTCAG-3′) and (R 5′-GGCTGCTGGCACGTAGTTAG-3′) were used to amplify the 16S-rRNA gene. This step was performed with a 25 μl final volume containing 0.4 μmol/L primers, 40 ng of chromosomal DNA, and the master mix (Ampliqon, Herlev, Denmark). The amplification reaction was completed using a thermocycler program that included 4 min at 96°C for initial denaturation, 30 s for denaturation for 30 cycles, 30 s at 48°C for annealing, and 45 s at 72°C for the first extension, with a final extension of 4 min at 72°C. The total volume of the reaction was 50 μl and the PCR product was visualized using 0.8% (w/v) agarose gel electrophoresis (14). The PCR product was sequenced by Macrogene Company (Korea) and was blasted on the NCBI and GenBank site (http://blast.ncbi.nlm.nih.gov/Blast.cgi).

Probiotic susceptibility to seven high-consumption antibiotics used in aquaculture, including oxytetracycline, tetracycline, amoxicillin, ampicillin, erythromycin, sulphonamides, and oxolinic acid, was assessed using a disc diffusion approach. The isolate was cultured in Mueller-Hinton agar plates, and the antibiotic disks were placed on plates with the use of sterilized forceps and then incubated at 37°C for 18–24 h (20). A digital caliper was used to measure the sizes of the clear zones around disks (21, 22) and the isolated strains were classified as sensitive, intermediate, and resistant based on the size of the clear zone (23).

An agar diffusion well method was applied to investigate the antibacterial activity of probiotic cells against the five most relevant pathogens likely to be found in aquatic farms such as *Salmonella enterica* (ATCC 9115), *Streptococcus agalactiae* (ATCC 13813), *Streptococcus iniae* (ATCC 29178), *Yersinia ruckeri* (PTCC 1888), and *Clostridium botulinum* (ATCC 3502) and incubated overnight in MRS broth medium at 37°C and the isolate was centrifuged (Hermle Z 36 HK, Germany) for 10 min at 6,000 × g (24).

The supernatant was filtered using a 0.2 μm filter and 100 μl of filtered neutralized supernatant was added to the 7 mm diameter wells created on a Mueller-Hinton agar plate, which was pre-inoculated with the above-mentioned indicator pathogens. The 8 cm agar plates were incubated overnight at 37°C and the clear zone was studied to evaluate the positive antimicrobial activity of isolated metabolites against the pathogens (25, 26). The probiotic susceptibility and resistance were evaluated under acidity of pH 3.0 and alkalinity using bile salt (0.5% w/v oxgall). The pH was set to 8 and the temperature was set to 37°C, then the culture medium was centrifuged (6,000 × g for 3 min) to replicate the digesting conditions in fish. These cell plates were re-suspended for 2 h by gentle agitation and the survival rates were calculated by the equation described by Haghshenas et al. (27).

#### Harvesting and inoculation of probiotic cell

The *E. faecium* cells were grown and amplified in 200 ml MRS medium for 18 h, at 37°C, under anaerobic conditions in an anaerobic jar (Mitsubishi Inc. USA) that contains an aerobic gas generation kits (AnaeroPack). The amplified cells were harvested by centrifuging at 18,000 × g for 20 min, at 4°C, washed, and re-suspended in 10 ml sterile phosphate buffer saline (PBS, Merck) at a pH of 7.2. Before usage, the concentrated probiotic cells were counted in MRS agar to the desired concentration (1 × 10^9^ log CFU mL^−1^) using the pour plate technique. In the microencapsulation procedure, equal quantities of the probiotic cell population were segmented and then combined with various biopolymers and prebiotic mixes.

#### Preparation of fenugreek gel powder

The Fk gel (Atarak herbal medicines online sales site (https://attarak.com), Iran, Product ID: 441001) was extracted according to Mandal et al. (28), with some modifications, in which 100 g of ground Fk seeds were soaked in 500 ml of distilled water for 1 h at a pH of 8, at 68°C and continuously stirred. A homogenous gel was centrifuged for 30 min at 12,000 × g to separate the gel phase, washed twice with distilled water, and applied in a microencapsulation matrix after being dried to a powder (by keeping at room temperature) (28).

#### Purification of Persian gum

The gel obtained from PG (Atarak herbal medicines online sales site (https://attarak.com), Iran, Product ID: 901291) was purified according to Simas-Tosin et al. (29), with some modifications. In brief, 30 g of dry PG was dispersed in 1,000 ml of distilled water at 70°C, pH 8, and was allowed to dissolve slowly over 12 h. The impurities were precipitated by high-speed centrifugation, at 18,000 × g for 20 min, to remove insoluble residues and the purified gel was dried overnight in the oven at 40°C, and the resulting powder was applied in the microencapsulation process according to Simas-Tosin et al. (29).

#### Microencapsulation of *E. faecium*

The *E. faecium* cells were microencapsulated by a modified extrusion method using ALG-PG with various prebiotic [FOS (Sigma-Aldrich, Germany, MDL number: MFCD 00677049) and Fk] concentrations. The un-microencapsulated cells and ALG-encapsulated cells (ALG) were used as the control. The PG gel, sodium ALG, FOS, and Fk were autoclaved (121°C for 20 min) before the microencapsulation step, then *E. faecium* cells [10% (w/v)] were suspended in 5 ml of FOS and Fk (0, 1, 1.5, and 2%) solutions and mixed with 10 ml of PG [0 and 0.5% (w/v)] and 10 ml of sodium ALG [0, 1.5, and 2% (w/v)] stock solutions to achieve microencapsulated probiotic cells (10^10^ CFU g^−1^).

The final prebiotic concentrations were 0, 0.2, 0.3, and 0.4%. A 5 ml sample of the gel solutions was stirred and mixed for 30 min to produce homogeneous mixtures, which were extruded through a 21-gauge nozzle in 300 ml sterile CaCl_2_ (0.5 M) to create the microencapsulated beads. The beads were filtered (using Whatman Paper No. 1) and rinsed twice with sterile water before being used for subsequent experiments and then stored in peptone solution [0.1% (w/v)] at 4°C, according to Haghshenas et al. (16).

#### Water activity, moisture content, and encapsulation efficiency (EE) of beads

At a constant temperature of 24 ± 0.5°C, the water activity of the beads was measured using a water activity meter (Dewpoint, USA) The moisture content of the powdered microencapsulated beads was also evaluated by drying the samples for 12 h at 105°C (26). The efficacy of encapsulation was determined using 100 mg of microencapsulated beads that were dissolved in 20 ml PBS at pH 7.2, and 37°C for 60 min, then the viable cells were serially diluted and counted as CFU per gram on MRS agar using the pour plate technique and calculated according to the following equation:


$$\begin{array}{l} \text{Encapsulation efficiency }\left(\text{EE}\right)\;=\;\left({\text{log}}_{10}\;{\text{M}/\text{log}}_{10}\;{\text{M}}_{0}\right)\;\times 100, \end{array}$$

Where *M* denotes the number of viable bacteria cells entrapped in the beads and Mo defines the free viable cells before microencapsulation, the data were expressed as the mean of three counts ± standard deviation (30).

#### Cell viability of *E. faecium* under fish simulated digestive conditions

Under fish simulated digestion circumstances, the protective effectiveness of beads and the cell viability of microencapsulated bacteria were assessed using 100 mg of each blend formulation, which were incubated individually by gentle stirring at 100 rpm in 20 ml of simulated gastric juices (pH 1.4 at 37°C) and intestinal juice (0.5% w/v oxgall, pH 8 at 37°C) for 0, 30, 60, 90, and 120 min. The beads then were disintegrated in 10 ml PBS (pH 7.2) and viable cells were counted by the pour plate technique according to the following calculation: Cell viability (%) = (log CFU g^−1^ cells after disintegrating/log CFUg^−1^ cells before disintegrate) × 100. In this formula, CFU displays the number of colony-forming units on MRS agar by the pour plate technique according to Haghshenas et al. (27).

#### Storage stability of microencapsulated *E. faecium* in feed pellets

The storage stability of microencapsulated and non-microencapsulated probiotic cells was examined (Dindings, Malayan Flour Mills, Berhad, Malaysia) over an 8 week storage period, and the viability of bacteria was measured weekly at 0, 7, 14, 21, 28, 35, 42, 49, and 56 d of storage at 25°C. The storage stability assay was completed using 5 g feed pellets that contained 500 mg of microencapsulated cells dissolved in 50 ml sodium citrate solution (50 mM) at room temperature and then gently stirred at 100 rpm and a pH of 8. Using a saline solution, the liberated cells were serially diluted 10 times, then 20 μl of the diluted solution was used to calculate the storage stability (%) of the cells following their anaerobic growth at 37°C for 24 h, using the pour plate method according to Haghshenas et al. (31). The storage stability was measured according to the following calculation: Storage stability (%) = (log CFU g^−1^ cells after storage time/log CFU g^−1^ cells before storage time) × 100. In this formula, CFU displays the number of colony-forming units on MRS agar by the pour plate technique.

#### Release assay of microencapsulated *E. faecium*

Using one gram of each microencapsulated bead, a release assay was performed in the microencapsulated *E. faecium* placed in 200 ml of simulated fish intestine solution containing digestive enzymes lipases, proteases, and carbohydrases, incubated (with gentle stirring at 100 rpm) at pH of 8 and temperature of 37°C. For a period of 12 h, samples were obtained at 1-h intervals and the released probiotic cells were counted using a pour plate technique according to Mandal et al. (28). The release rate (%) = (log CFU g^−1^ probiotic cells after release time/log CFU g^−1^ probiotic cells before release time) × 100. In this formula, CFU displays the number of colony-forming units on MRS agar by the pour plate technique.

### Phase 2: *In vivo* challenge study

#### Fish growth conditions

Red hybrid tilapia fish were purchased from a commercial farm in the research center of University Putra Malaysia (UPM). They were weighed (mean 50 ± 0.55 g) at the juvenile stage and transported to two-tone (5.67 m^3^) fiberglass tanks at the research center of UPM, Puchong, Malaysia where they were acclimatized for 2 weeks. The adaptation period was performed slowly after feeding with commercial food pellets (Dindings, Malayan Flour Mills, Berhad, Malaysia) for twice a day (5% of the body weight). During this period, the water was maintained at a pH between 7.2 and 8, temperature of 27 (± 2)°C, hardness of 75–100 mg L^−1^, dissolved oxygen of 7–8 mg L^−1^, and an ammonia concentration of <0.1 mg L^−1^, which was renewed at an equivalent of 10% of the water daily to remove waste feed and fecal matter.

#### Experimental treatments and design

In a glass aquarium rearing system, the 90-days *in vivo* investigation was completed. Three hundred and thirty healthy fish were divided into glass tanks, 10 fish in each tank with three replicates. Seven experimental treatments were formulated using alginate-Persian Gum (PG) with various prebiotic [fructooligosaccharides (FOS) and fenugreek (Fk)] concentrations. Meanwhile, un-microencapsulated cells and alginate-encapsulated cells (ALG) were used as the control.

Alginate or its mixture with other gums at concentrations <1% (w/v) due to low viscosity and lack of crosslinking sites does not create uniform and spherical microencapsulated beads. On the other hand, extrusion of combined hydrogels at concentrations above 2% (w/v) due to high viscosity is difficult and impossible. Therefore, *E. faecium* ABRIINW.N7 cells [10% (w/v)] were suspended in 5 ml of FOS and Fk (0, 1.0, 1.5, and 2%) solutions and then mixed with 10 ml of PG [0.5% (w/v)] and 10 ml of sodium alginate [1.5% (w/v)] stock solutions. The final prebiotic concentrations were 0, 0.2, 0.3, and 0.4% respectively. For diet preparation, 900-g commercial extruded food pellets (Dindings, Malayan Flour Mills, Berhad, Malaysia) were mixed with 100-g microencapsulated probiotic cells during a 1-h drying phase under constant airflow. After final drying, food pellets were vacuum-packaged into germfree plastic bags and stored at 25°C.

The diets for the fish were as follows: negative control treatment (CON) without *S. agalactiae* challenge; positive control treatment, (CON^+^) with *S. agalactiae* challenge; Free cell with *S. agalactiae* challenge in a free cell format; ALG with *S. agalactiae* challenge and 0.8% (w/v) Alginate; microencapsulated *E. faecium* probiotic treatment incorporated with FOS and Fk (F1–F7) *S. agalactiae* strain was isolated in Malaysia from suspected fish from the two farms in (i) 40 fish from Rawang, Selangor and (ii) 60 fish from Tasik Kenyir, Kuala Berang, Terengganu, Malaysia in 2014. Strain was identified phenotypically and biochemically by using API 20 streps and 16S rRNA technique. *Streptococcus agalactiae* glycerol stock was sub-cultured in blood agar. Few colonies were sub-cultured into Brain heart infusion broth (BHIB; Merk, Germany). To enhance the virulence of bacteria, 1 ml of *S. agalactiae* with a high concentration (10^9^ CFU ml^−1^) was injected (intraperitoneal) to red hybrid tilapias. Fish showing signs of streptococcosis were euthanized to re-isolate the *S. agalactiae*. Then, the confirmed isolates were sub-cultured on blood agar before inoculating into the BHIB. Sixty colonies were sub-cultured into the BHIB. Afterward, 10-fold serial dilution and colony counting were utilized to determine the bacterial concentration. This virulent bacterial inoculum was immediately used for the challenge. The fish were given an intraperitoneal injection of 0.1 ml of *S. agalactiae* (1.6 × 10^8^ CFU ml^−1^) and one group was selected as the control group and injected with 0.1 ml of PBS. The injection was done by 1 ml insulin syringe. After acclimatizing the fish with virulent *S. agalactiae* for 2 weeks and onset of the streptococcosis signs in treated groups, 90-day feeding with the mentioned formulations was started. The aquariums were aerated with freshwater at 27 ± 2°C and equipped with top filters and were changed twice a week along with water siphoning to remove the feces.

For food pellet preparation (200 kg), 900-g commercial extruded food pellets (Dindings, Malayan Flour Mills, Berhad, Malaysia) were mixed with 100 g microencapsulated probiotic cells (10^10^ CFU g^−1^) and dried for 1 h under constant airflow to achieve 10^9^ CFU g^−1^. The aquariums were aerated with fresh water, and the fish were fed twice a day (5% of the body weight) with probiotic food pellets containing different concentrations of hydrogels.

After the experiment, the treated fish were killed to re-isolate the candidate probiotic and prove the colonizing of *E. faecium* ABRIINW.N7 in the gastrointestinal tract of fish. Moreover, water in glass aquariums was assessed for the presence of *E. faecium* ABRIINW.N7 in the environment.

#### Anti-pathogenicity against streptococcus agalactiae and histopathological assay

To cover the taste of the various microencapsulation compounds, microencapsulated and non-microencapsulated probiotic cells were incorporated in a prevalent and palatable commercial fish pellet (Dindings, Malayan Flour Mills, Berhad, Malaysia) in a ratio of 1: 10. Fish were anesthetized by adding 0.1 ml clove oil (270 ppm) per lot and their weight was recorded weekly. The weight gain (WG), daily weight gain (DWG), relative growth rate (RGR), percent weight gain (PWG), and specific growth rate (SGR) were calculated according to Ng et al. (32) but no significant differences (*P* ≤ 0.05) were observed in different groups.

During 90 days, the fish were screened for any abnormal behavior, clinical signs, survival post-injection, and dead fish were assessed for pathogens isolated from the kidney, liver, eye, and brain using loops. The samples were cultured on brain heart infusion broth (BHIB; Merk, Germany) and incubated at 30°C for 24 h to determine if mortality was caused by *Streptococcus* species. Histopathological examinations were performed on tissue samples taken from the fish's liver, spleen, kidney, brain, and eye. The tissues were soaked in 10% formalin for at least 24 h before being inserted into the cassette. All the samples of organs and guts were placed in processing machines with various concentrations of ethanol and melted paraffin. Then, the tissues were embedded into paraffin blocks and allowed to freeze. The samples were sectioned with a Jung Multicut microtome (Leica, Germany) and located on the slides. Finally, the slides were subjected to routine Harris' Hematoxylin and Eosin (H&E) staining, and the slides were checked under a microscope (Olympus, Japan). The survival rate was revealed according to the following formula (33):


$$\begin{array}{l} Survival\;Rate\;\left(\%\right)\;=\;\frac{\text{Number of live fish at the end of test}}{\text{Primary number of live fish}}\\ \;\times 100. \end{array}$$

Meanwhile, to calculate the concentration of candidate probiotic strain in the gastrointestinal tract of fish and water in glass aquariums, under sterile conditions, the fish gut (1 g) was dissected out and homogenized with 10 ml of normal saline. The homogenate gut and aquarium water samples (1 ml) were kept in a boiling water bath at 85°C for 15 min to remove fungal contaminants. The homogenate was serially diluted and pour plated onto MRS agar medium. Plates were incubated under anaerobic conditions at 37°C for 48 h. Individual colonies were picked up and purified by the streaking method in a fresh MRS agar medium. All the isolates were identified by PCR with strain-specific primers and stored at −80°C in nutrient broth supplemented with 40% glycerol.

### Statistical analysis

The experiments were treated as completely randomized design with three replications for each experimental group. Normality test of data was performed using the Shapiro–Wilk test. The data were then analyzed using ANOVA (SPSS software) and Duncan test and means with *P* < 0.05 was considered as the significant differences.

## Results and discussion

### Molecular identification and strain characterization of *E. faecium*

The sequencing of the 16S-rRNA PCR-amplified fragment (1,500 bp) was performed and compared with the sequences deposited in GenBank. The strain isolated from the ewe colostrum was identified with 99%−100% homology as *E. faecium* (Accession number: MK367697). Following FAO/WHO guidelines, molecular identification of probiotic strains by 16S-rRNA sequencing (34) the threshold value for bacterial taxonomic studies was found ~97% similar to the approach of Nami et al. (35) and Deng et al. (36). The assessment of the probiotic properties of bacteria should be completed according to standards of *in vitro* experiments, including the susceptibility to antibiotics, anti-pathogenic activity, and resistance to acid and bile in the digestive tract. In this study, the *E. faecium* showed appropriate antibiotic susceptibility and acceptable anti-pathogenic activity (Table 1) and was sensitive to all seven antibiotics assessed and inhibited the growth of all five pathogens assessed, however, the tolerance to 0.3% bile salt concentrations and pH 3.0 was poor. Therefore, to compensate for this weakness, the viability of microencapsulation, using differing biopolymer-prebiotic formulations, was evaluated using *in vitro* assessment in this study.

**Table 1:** Antibiotic susceptibility of isolated *Enterococcus faecium* against the high consumption antibiotics performed by disk diffusion assay and antimicrobial activity of isolate against the pathogenic bacteria.

**Strain**	^*^ **Antibiotic susceptibility [clear zone (mm)]**						
	**Oxytetracycline** **(30** **μg)**	**Tetracycline** **(30** **μg)**	**Amoxicillin** **(25** **μg)**	**Ampicillin** **(10** **μg)**	**Erythromycin** **(15** **μg)**	**Sulphonamides** **(30** **μg)**	**Oxolinic acid** **(30** **μg)**
*E. faecium*	20.2 ± 2.3	21.4 ± 3.3	23.1 ± 2.6	18.2 ± 2.7	25.4 ± 3.1	16.2 ± 2.6	17.3 ± 2.4
**Strain**	^*^ **Antimicrobial activity [clear zone (mm)]**						
	***Streptococcus agalactiae***		***Salmonella enterica***	***Streptococcus iniae***		***Yersinia ruckeri***	***Clostridium botulinum***
*E. faecium*	19.2 ± 1.8		13.4 ± 1.6	18.3 ± 1.5		13.3 ± 1.7	14.4 ± 1.4

* All tests were performed in triplicate.

### Size determination water activity, moisture content, and encapsulation efficiency (EE) of beads

The average diameters (based on 50 beads) for alginate, alginate-PG, and alginate-PG blend with FOS and Fk were 790–980, 320–350, 360–410, and 540–670 μm, respectively (Table 2). The mean diameters of beads containing alginate-PG (F1) or alginate-PG blend with FOS (F2–F4) were significantly (*P* < 0.05) smaller than beads containing alginate-PG blend with Fk (F5–F7). Meanwhile, the mean diameters of beads containing lower concentration of FOS (F2 and F3) and Fk (F5 and F6) were significantly (*P* < 0.05) smaller than high concentration ones (F4 and F7) (Table 2).

**Table 2:** Compositions, size, water activity, moisture content (%), and encapsulation efficiency (%) of microencapsulated *Enterococcus faecium* with various gel and prebiotic concentrations.

**Formulation**	**Alginate** **(% w/v)**	**PG** **(% w/v)**	**Prebiotic (FOS)** **(% w/v)**	**Prebiotic (fenugreek)** **(% w/v)**	**Diameter (**μ**m)** **(*****n** =* **50)**	**Water activity**	**Moisture content** **(%)**	**Encapsulation efficiency** **(%)**
ALG	0.8	0	0	0	790–980	0.55 ± 0.002^*^	3.22 ± 0.04^*^	99.1 ± 0.7^*^
F1	0.6	0.2	0	0	320–350	0.48 ± 0.003^b^	3.25 ± 0.06^a^	98.8 ± 0.5^a^
F2	0.6	0.2	0.2	0	360–370	0.37 ± 0.001^c^	3.12 ± 0.03^a^	99.4 ± 0.9^a^
F3	0.6	0.2	0.3	0	360–380	0.36 ± 0.004^c^	3.29 ± 0.06^a^	99.6 ± 0.6^a^
F4	0.6	0.2	0.4	0	390–410	0.34 ± 0.005^c^	2.98 ± 0.02^a^	99.0 ± 0.4^a^
F5	0.6	0.2	0	0.2	540–580	0.27 ± 0.006^d^	3.18 ± 0.07^a^	98.6 ± 0.7^a^
F6	0.6	0.2	0	0.3	570–600	0.25 ± 0.007^d^	2.92 ± 0.05^a^	99.3 ± 0.8^a^
F7	0.6	0.2	0	0.4	640–670	0.24 ± 0.002^d^	3.08 ± 0.08^a^	99.5 ± 0.9^a^

Alginate-encapsulated cells [2% (w/v)] were used as control. F1–F7: various gel formulations. Values shown are means ± standard deviations (n = 3).

* Values followed by the same letters are not significantly different (P < 0.05). Statistical analysis of each formulation was done separately.

ALG, alginate-encapsulated cells; PG, Persian gum; FOS, fructooligosaccharides.

According to other researches, small size beads (10–40 μm) have been observed (37). But, in this study medium size beads with had no adverse effect on the structure and texture of food pellets have been produced (38). High variations in the size of beads can be due to the different composition and concentration of the polymers (39). The relatively larger mean diameter sizes of beads in alginate-PG + fenugreek formulations may alter the structure in texture sensitive dairy products such as cream (40), but there is no limitation on their application in fish industry such as probiotic food pellets. Since fenugreek polymer consists of D-mannan chains with D-galactopyranoyl side-chains structure, can anticipate that it exhibits higher viscosity value as compared to FOS. A possible explanation for successful larger sizes of bead with uniform spherical shapes in alginate-PG + fenugreek formulations. These results are consistent with other studies that demonstrate the reduced viscosity of supporting gels leading to smaller beads (16, 41).

IIn this study, the ALG-PG (F1) and control (ALG) formulations showed the greatest water activity value (*P* < 0.05) compared to the other formulated blends. The water activity levels for the ALG-PG + Fk (F5–F7) formulations, on the other hand, were lower than the other formulations (Table 2). High water activity lowers the viability of encapsulated probiotic cells, lowering the probiotic beads' long-term storage capacity (42). Gardiner et al. (43) and Eratte et al. (44) reported low water activity and dampness substance that matched our results during probiotic encapsulation. As a result of the low water activity and remaining water substance, dependable and storable encapsulation beads containing probiotic cells can be developed.

The moisture content of beads prepared in this study was below 3.29% (w/w) and there was no difference in the moisture content of the seven gels and control (ALG) formulations, which was consistent with previously published results (45). Other researchers observed low moisture content of prepared beads same as our results during microencapsulation. Further research has shown that low residual water content same as our results can improve the stability and storage capacity of probiotic-containing beads (8, 15).

In this investigation, there was no difference in encapsulation efficiency between the seven gel and the ALG as the control (Table 2) formulations, all of which showed a high encapsulation efficiency (>98.6%) that indicated the successful entrapment of viable probiotic cells within the beads (1–2 × 10^8^ CFU g^−1^) at the required site of impact. Similarly, several researchers have observed high rates of encapsulation efficiency, close to 100%, using various encapsulation techniques (40). The findings of this investigation demonstrated that encapsulation efficiency was unaffected by formulation, however other sources claimed that encapsulation effectiveness was impacted by polymer concentration and composition (46, 47).

### Cell viability of *E. faecium* under fish simulated digestive conditions

The unencapsulated *E. faecium* were very susceptible to simulated fish digestive conditions, according to our findings. Our results showed that the cell viability were low, changing from an initial cell count of 9.87 ± 0.02 to 3.85 ± 0.05 log CFU g^−1^ following incubation under harsh conditions, resulting in a survival rate of ~39% (Table 3). The reported cell viability was similar to results from other studies demonstrating a substantial loss of free probiotics cells in simulated digestion conditions (37, 40).

**Table 3:** Cell viability of microencapsulated *Enterococcus faecium* with various gel and prebiotic concentrations after incubation in simulated fish gastric juices (0.08 M HCl containing 0.2% NaCl, pH 1.4) for 0, 30, 60, 90, and 120 min and sequentially in stimulated fish intestinal juice containing (0.5% w/v oxgall, pH 8 at 37°C for 120 min).

**Formulation**	**Prebiotics**	**Con. (%)**	**Mean count of cells after incubation (log CFU g** ^−1^ **)**					**Cell viability (%)**
			**0 min**	**30 min**	**60 min**	**90 min**	**120 min**	
Un-microencapsulated cells	0	0	9.87 ± 0.02^a^	5.12 ± 0.04^b^	4.73 ± 0.05^b^	4.18 ± 0.01^c^	3.85 ± 0.05^c^	39 ± 0.7^f^
ALG	0	0	9.76 ± 0.04^a^	5.04 ± 0.01^b^	4.77 ± 0.06^c^	4.59 ± 0.07^c^	4.39 ± 0.06^d^	45 ± 0.3^e^
F1	0	0	9.69 ± 0.02^a^	6.23 ± 0.04^b^	5.94 ± 0.03^c^	5.46 ± 0.08^d^	5.14 ± 0.01^e^	53 ± 0.4^d^
F2	FOS	0.2	9.92 ± 0.07^a^	7.18 ± 0.03^b^	6.74 ± 0.02^c^	6.51 ± 0.04^c^	6.05 ± 0.04^d^	61 ± 0.2^c^
F3	FOS	0.3	9.79 ± 0.03^a^	7.42 ± 0.01^b^	7.09 ± 0.09^c^	6.58 ± 0.04^d^	6.17 ± 0.03^e^	63 ± 0.2^c^
F4	FOS	0.4	9.94 ± 0.04^a^	7.58 ± 0.03^b^	7.26 ± 0.02^b^	6.89 ± 0.07^c^	6.36 ± 0.02^d^	64 ± 0.8^c^
F5	FK	0.2	9.57 ± 0.05^a^	7.97 ± 0.04^b^	7.56 ± 0.03^c^	7.36 ± 0.04^c^	6.99 ± 0.07^d^	73 ± 0.6^b^
F6	FK	0.3	9.91 ± 0.06^a^	8.12 ± 0.01^b^	7.91 ± 0.04^c^	7.74 ± 0.07^c^	7.43 ± 0.05^d^	75 ± 0.3^b^
F7	FK	0.4	9.83 ± 0.01^a^	8.59 ± 0.02^b^	8.33 ± 0.06^c^	8.12 ± 0.05^d^	7.96 ± 0.04^d^	81 ± 0.7^a^^*^

Alginate-encapsulated cells [0.8% (w/v)] were used as control. F1–F7: various gel formulations. Values shown are means ± standard deviations (n = 3).

* Values followed by the same letters are not significantly different (P < 0.05). Statistical analysis of each formulation was done separately.

ALG, alginate-encapsulated cells; FOS, fructooligosaccharides; Con, concentration; SR, survival rate.

The cell viability of all seven gel formulations was higher than that of ALG-encapsulated beads (control) following the exposure to simulated fish digestive conditions, however, the highest microencapsulated cell survival rates were observed in the F5, F6, and F7 (Table 3). The *E. faecium* viability rates in microencapsulated in ALG-PG blend with 1, 1.5, and 2% Fk were 73, 75, and 81% respectively and the survival rate for ALG-PG blend with 0.4% Fk (F7) was the highest in this study, showing a 1.87 log decrease in the cell CFU counts within the first 2 h of incubation, while other blends showed a continuous decrease of between 2.48 and 5.37 log in the cell CFU. The cell viability of potential probiotic *E. faecium* in un-microencapsulated cells decreased from 9.87 ± 0.02 to 3.85 ± 0.05 (SR = 39), while the cell viability of this potential probiotic microencapsulated with 0.8% FK dropped from 9.83 ± 0.01 to 7.96 ± 0.04 (SR = 81%). Furthermore, the highest cell viability of *E. faecium* microencapsulated with 0.8% FOS was 64% (decreased from 9.94 ± 0.04 to 6.99 ± 0.07). Similarly, well-protected formulations, with the ability to survive the harsh digestive condition, have been reported when psyllium (10), whey protein (48), and milk (49) were incorporated with ALG. Combining FOS and Fk with an ALG-PG combination boosted probiotic cell viability (by 22–42 percentage points), and increasing prebiotic content from 0.2 to 0.4 percent (% w/v) increased cell vitality even more, probably owing to prebiotics' protective and nutritive properties. In a simulated digestive condition, Fk, on the other hand, had a larger positive effect (by 34–42 percentage points) than FOS (by 22–25 percentage points).

Fk's great protective ability can be attributed to the higher density of beads produced by its robust structure (8). The binding of glucuronic acid to divalent cations is primarily responsible for the crosslinking of ALG molecules. The molecular weight and chemical composition of ALG-biopolymer membranes affect their stability and permeability (50). As a result, combining ALG with flexible biopolymers like Fk enhances the strength of synthetic blends. The stability of the ALG-biopolymer is determined by the precursor structure, biopolymer molar ratio, and addition sequence (51).

### Storage stability of microencapsulated *E. faecium* in food pellet

**Figure 1:**
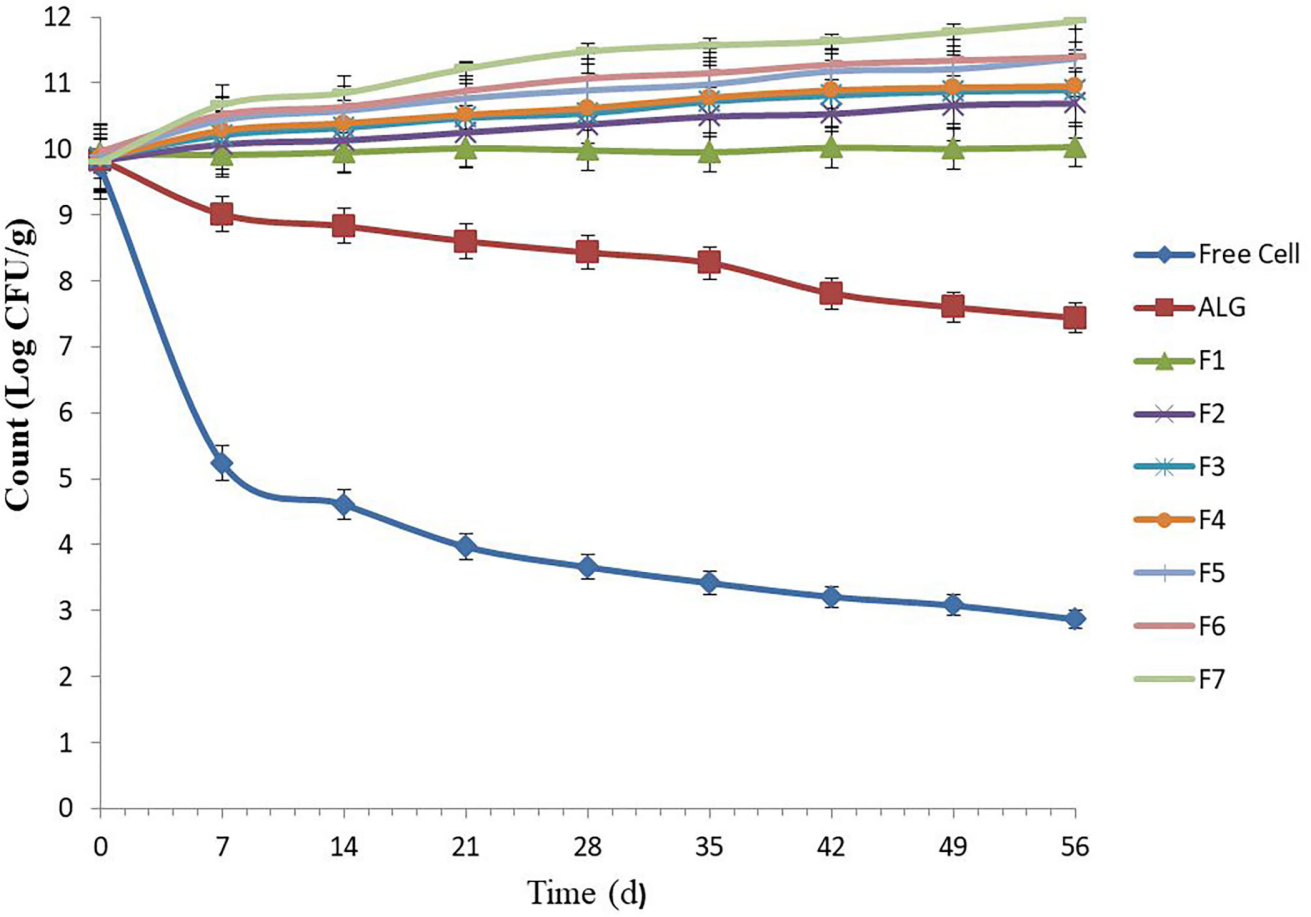
Number of free and microencapsulated *E. faecium* with different gel and prebiotic formulations during 8 weeks storage in food pellet at 25°C. Alginate-encapsulated cells [0.8% (w/v)] were used as control. F1: ALG (0.6%) + PG (0.2%); F2: ALG (0.6%) + PG (0.2%) + FOS (0.2%); F3: ALG (0.6%) + PG (0.2%) + FOS (0.3%); F4: ALG (0.6%) + PG (0.2%) + FOS (0.4%); F5: ALG (0.6%) + PG (0.2%) + Fk (0.2%); F6: ALG (0.6%) + PG (0.2%) + Fk (0.3%); F7: ALG (0.6%) + PG (0.2%) + Fk (0.4%). Values shown are means ± standard deviations (*n* = 3).

Several food products can be used as probiotic carriers, such as pelleted fish feed. These carriers can be stored for 6–8 weeks at room temperature (25°C) (16). The free *E. faecium* cell viability in un-microencapsulated *E. faecium* decreased from 9.73 to 2.87 log CFU g^−1^ during the whole 7-week storage period, which was greatest during the first week followed by a gentle consistent decline, likely due to a temperature shock (25°C) for the cells during the first week, followed by an adaptation process (Figure 1). A similar decrease trend was observed by Haghshenas et al. (16), in which the survival rate of free *E. durans* cell count in yogurt lowered from 9.52 to 2.83 log CFU g^−1^ following 1 month of storage.

Our results were consistent with other researchers and showed that *E. faecium* microencapsulated in ALG (control) and all seven gel formulations had high storage stability (10, 19). Previously, the low-temperature storage stability (25°C) of encapsulated probiotics in ALG-gum Arabic (19), ALG-chitosan (10), and ALG-psyllium (10) was assessed. In this study, the ALG-PG (F1) and ALG-PG blended with FOS (F2–F3) showed high protection, with a 0.10 to 1.09 log increase in CFU g^−1^, while an excellent cell viability during storage (>100%) was found when ALG-PG blended with 0.4% FOS (F4) and ALG-PG + Fk formulations (F5–F7) were evaluated. Moreover, the gel formulated with greater Fk concentrations (F7) in this study, showed a greater protective capacity compared to lower Fk concentrations (F5 and F6; Figure 1), due to greater Fk (0.4%) concentration forming a dense, strong membrane and growth-stimulating activity of Fk, however, the extrusion of highly concentrated blends are difficult to press through the nozzle gage, which lowers the encapsulation efficiency.

### Release assay of microencapsulated *E. faecium*

Effective microencapsulation of probiotics into food is required to promote the health condition of animals. To achieve this goal, the probiotic cells must be released in sufficient quantity and within an appropriate time frame. Thus, the time-dependent release of probiotic cells from the microencapsulation beads within the simulated intestine solution is critical (28) and, in this study, an initial number (1 × 10^7^ CFU g^−1^) of *E. faecium* was selected for release assay. However, previous researches have indicated that the concentration and composition of polymers used in the microencapsulation process influence the release of cells from the beads (51).

**Figure 2:**
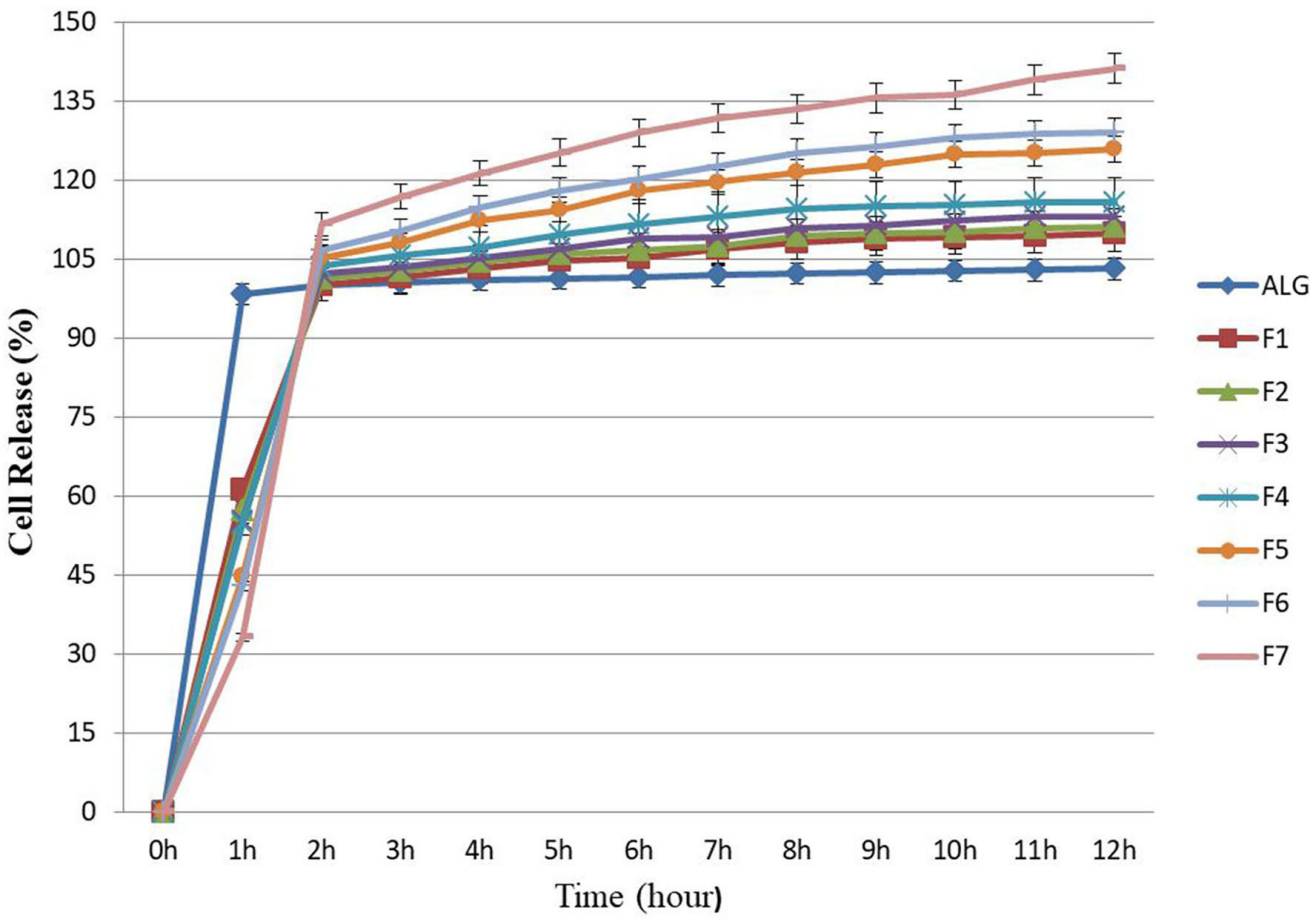
Releasing rates of microencapsulated *E. faecium* with different gel formulations at simulated intestine pH solution containing digestive enzymes for each hour (up to hour 12). ALG: alginate-encapsulated cells (control). F1: ALG (0.6%) + PG (0.2%); F2: ALG (0.6%) + PG (0.2%) + FOS (0.2%); F3: ALG (0.6%) + PG (0.2%) + FOS (0.3%); F4: ALG (0.6%) + PG (0.2%) + FOS (0.4%); F5: ALG (0.6%) + PG (0.2%) + Fk (0.2%); F6: ALG (0.6%) + PG (0.2%) + Fk (0.3%); F7: ALG (0.6%) + PG (0.2%) + Fk (0.4%). Values shown are means ± standard deviations (*n* = 3).

In this study the Log CFU g^−1^, for released *E. faecium* from ALG blend (control), was stable (7–7.2) and there were no significant changes in the rate of bacterial growth (Figure 2), which was similar to results previously reported by Mandal et al. (28) and Nami et al. (19) in terms of the sustained and continuous release of cells from ALG. Once the cell release was complete, there were significant additive release rates observed from the prepared formulations (F1–F7), resulting in a greater amount of release due to the growth-stimulating effects of PG, FOS, and Fk. In this study, the microencapsulation of *E. faecium* with ALG-PG or Fk (F5–F7) could release between 33 and 44% of the probiotic cells from encapsulated beads after 1 h of incubation and was fully released after 2 h. The number of bacterial cells (Log CFU g^−1^) for F5, F6, and F7 formulations increased from 7 to 9.9, which was greater than the release rates from the other formulations (7–8.1), while a greater concentration of Fk (0.4%) (F7) lowered the rate of probiotic *E. faecium* cell release (33%) within the first hour. The full release was completed after 2 h (Figure 2), showing how the addition of Fk to the ALG-PG blend lowered the rate of probiotic cell release from the beads. Similar results have been observed previously (8, 16), which were probably due to a more dense membrane on the beads that were covered by the rigid structure of Fk.

In this study, about 54 and 61% of the probiotic *E. faecium* cells were released from the ALG-PG (F1) and ALG-PG + FOS blends (F2–F4) within 1 h, while the full release was completed after 2 h. The greater early rate of release compared with Fk in this study was probably due to the erosion of loose networks in formulations.

### Anti-pathogenicity against *Streptococcus agalactiae* and histopathological assay

**Figure 3:**
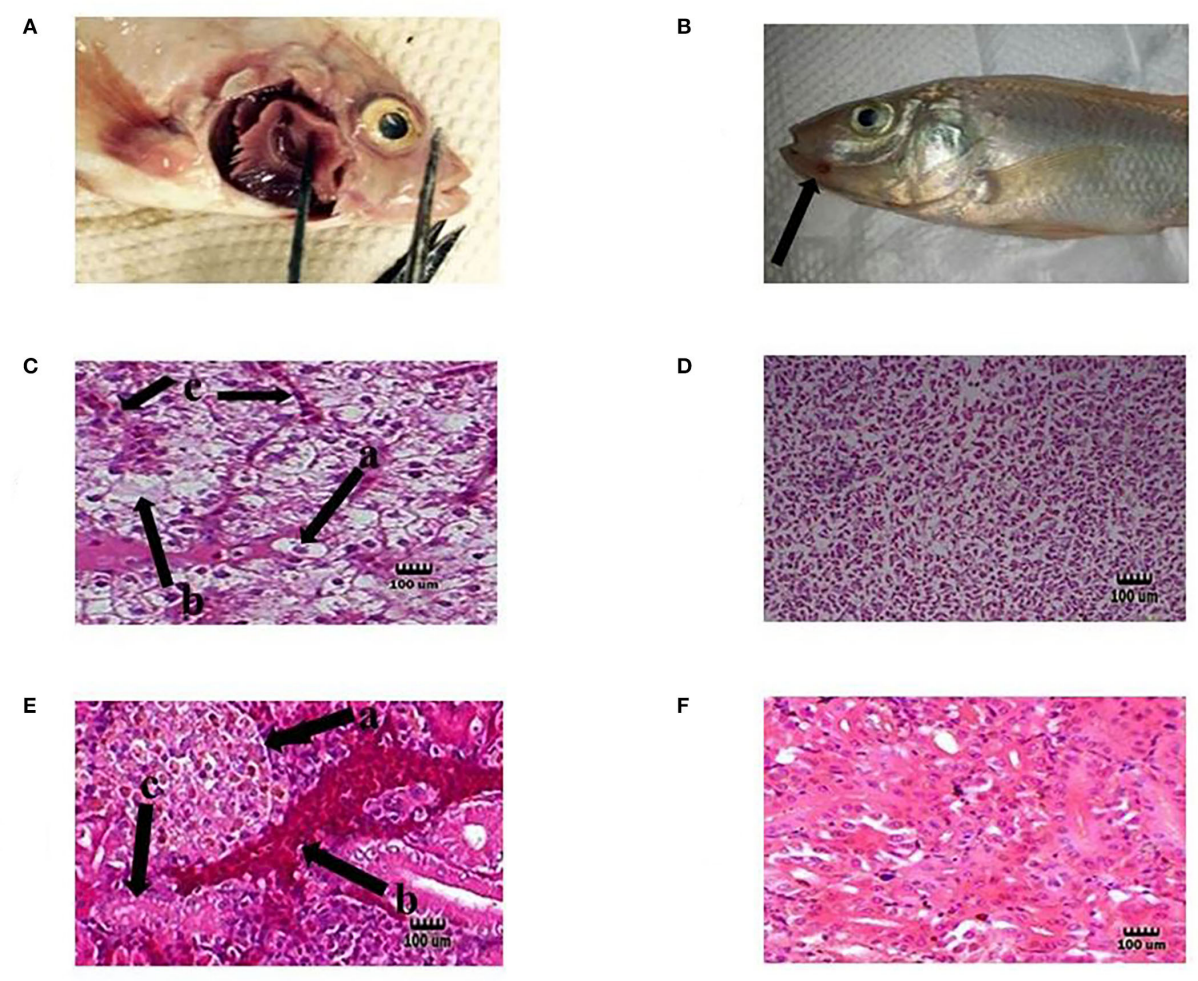
Representative of the fish in each test group. Hemorrhage on the base of the pectoral fin **(A)** and near the mouth **(B)** in the infected tilapia (black arrow). Degeneration hepatocytes i.e., swelling [**C** (a)], necrosis [**C** (b)], and congestion blood vessels [**C** (c)] in the liver of infected tilapia compared with normal liver tissue in the probiotic treated group without any infection **(D)**. Presence of corpuscles of stannius [**E** (a)], congestion in renal vessel [**E** (b)], and degeneration of tubular cells [**E** (c)] in the kidney of infected fish compared with normal kidney tissue in the probiotic treated group without any infection by *S. agalactiae* **(F)**. H&E, Mag. 1,000×. (Bar = 100 μm).

In this study, tilapia fish infected with *S. agalactiae* showed hemorrhage, red skin, particularly around the anus and eyes, exophthalmia, cloudy eyes, and erratic swimming movement. In addition, some bleeding and ulcers near the mouth were detected (Figures 3A,B), as described by previous researchers (16, 52). The tissue samples collected from the eye, kidney, liver, brain, spleen, and skin of infected fish showed clinical signs of Streptococcosis and histopathological changes including hemorrhage and congestion of blood vessels in the brain, eye, kidney, liver, and spleen. Melanomacrophage centers in the kidney, liver, and spleen were observed.

Moreover, dissolution, degeneration of some tubules, necrosis of tubular cells, glomeruli degeneration, attachment to bowman capsule, and congestion of renal vessels were observed in the kidney. Liver changes included swelling, degeneration, and necrosis of hepatocytes, and nuclear pyknosis (Figures 3C,E). Also, the organs of the fish in the probiotic treated and control group with no signs of infection were chosen for histopathology test as normal organs (Figures 3D,F). The clinical indications found were identical to those seen in earlier research, indicating that the tilapia fish were infected with streptococcal pathogenic agents (*S. agalactiae*) (53). The infected organs were also sampled, and the source of infection was determined to be *S. agalactiae* bacteria following biochemical and molecular identification. Probiotics boosted fish survival rates when exposed to pathogenic *Aeromonas hydrophila, Flavobacterium psychrophilum, Streptococcus iniae, Vibrio harveyi, and Pseudomonas fluorescens*, according to previous studies (54–56).

Results showed no significant effect on the growth performance was observed after 90 days of feeding with food pellets containing *E. faecium* microencapsulated with various gel and prebiotic concentrations (Table 2). The same results were documented by different researchers (32). On the other hand, other researches proved that feeding with probiotic-containing food pellets advanced the growth performance of fish (33). The survival rates of the treated red hybrid tilapia with free (17 ± 1.25%) or probiotic cells (24 ± 2.05%−63 ± 1.75%) were higher than those of the infected fish that were not treated with probiotics (CON^+^; 4 ±1.15%). Furthermore, the ALG-PG + 0.4% Fk formulation (F7) had a relatively high survival rate (63 ± 1.75%) while the control group injected with PBS (CON) had no mortality during the trial period (Table 4).

**Table 4:** Growth performance, mortality and survival rates of fish infected with *Streptococcus agalactiae* (1.6 × 10^8^ CFU ml^−1^) after 90 days of feeding with food pellets containing *Enterococcus faecium* microencapsulated with various gel and prebiotic concentrations.

**Formulation**	**Alginate** **(% w/v)**	**PG** **(% w/v)**	**Prebiotic (FOS)** **(% w/v)**	**Prebiotic (FK)** **(% w/v)**	**Probiotic strain**	**Pathogen strain**	**WG** **(g)**	**DWG** **(g)**	**RGR** **(%/day)**	**PWG** **(%)**	**SGR** **(%)**	**Fish mortality**	**Survival rates** **(%)**
CON	0	0	0	0	*_*	_	90.9 ± 3.6	1.01	2.1 ± 0.3	181.8 ± 7.3	1.15 ± 0.02	0	100.00 ± 0.0^h^
CON^+^	0	0	0	0	*_*	*S. agalactiae*	88.2 ± 5.3	0.98	1.8 ± 0.5	176.4 ± 4.6	1.13 ± 0.07	29/30	3.33 ± 1.00^g^
Free Cell	0	0	0	0	*E. faecium*	*S. agalactiae*	91.8 ± 1.6	1.02	2.2 ± 0.2	183.6 ± 2.7	1.16 ± 0.05	25/30	16.66 ± 2.00^f^
ALG	0.8	0	0	0	*E. faecium*	*S. agalactiae*	92.3 ± 6.5	1.02	2.3 ± 0.4	183.9 ± 8.4	1.17 ± 0.09	23/30	23.33 ± 2.00^e^
F1	0.6	0.2	0	0	*E. faecium*	*S. agalactiae*	92.7 ± 4.6	1.03	2.3 ± 0.1	185.4 ± 9.3	1.18 ± 0.03	20/30	33.33 ± 0.00^d^
F2	0.6	0.2	0.2	0	*E. faecium*	*S. agalactiae*	87.3 ± 7.2	0.97	1.8 ± 0.7	174.6 ± 5.1	1.12 ± 0.06	18/30	40.00 ± 3.00^c^
F3	0.6	0.2	0.3	0	*E. faecium*	*S. agalactiae*	89.1 ± 5.5	0.99	2.0 ± 0.4	178.2 ± 8.6	1.13 ± 0.05	18/30	40.00 ± 1.00^c^
F4	0.6	0.2	0.4	0	*E. faecium*	*S. agalactiae*	90.6 ± 3.7	1.00	2.1 ± 0.8	180.9 ± 4.1	1.14 ± 0.08	17/30	43.33 ± 0.00^c^
F5	0.6	0.2	0	0.2	*E. faecium*	*S. agalactiae*	88.8 ± 2.9	0.98	1.9 ± 0.5	176.7 ± 9.4	1.13 ± 0.03	15/30	50.00 ± 1.00^b^
F6	0.6	0.2	0	0.3	*E. faecium*	*S. agalactiae*	93.6 ± 6.2	1.04	2.4 ± 0.1	187.2 ± 5.9	1.18 ± 0.04	15/30	50.00 ± 3.00^b^
F7	0.6	0.2	0	0.4	*E. faecium*	*S. agalactiae*	90.7 ± 7.3	1.01	2.2 ± 0.7	181.2 ± 9.8	1.15 ± 0.09	11/30	63.33 ± 2.00^*^

The control group (CON) was injected with 0.1 ml of phosphate-buffered saline (PBS, pH 7.4). CON^+^ group injected with 1.6 × 10^8^ CFU ml^−1^ of S. agalactiae without probiotic treatment. F1–F7: various gel formulations. Values shown are means ± standard deviations (n = 3).

* Values followed by the same letters are not significantly different (P < 0.05). Statistical analysis of each formulation was done separately.

Free Cell, un-encapsulated probiotic cells; ALG, alginate-encapsulated probiotic cells; PG, Persian gum; FOS, fructooligosaccharides.

WG (g): weight gain: [final wt.(g) – initial wt.(g)].

DWG (g): daily weight gain.

RGR (%/day): relative growth rate: [(final wt.(g) – initial wt.(g))/initial wt.(g) × duration of study (days)] × 100.

PWG (%): percent weight gain: [(final wt.(g) – initial wt.(g))/initial wt.(g)] × 100.

SGR (%): specific growth rate: [ln(final wt.(g)) – ln(initial wt.(g))/days] × 100.

Other researchers found that complex dietary probiotics containing *Bacillus* and *Pediococcus spp*. had low anti-pathogenic activity against *S. agalactiae* in red hybrid tilapia (57); however, according to the present study, probiotic *E. faecium* isolated from ewe colostrum demonstrated excellent resistance to *S. agalactiae* in red hybrid tilapia for the first time. Other studies, on the other hand, demonstrated that *B. subtilis*-containing diets had no anti-pathogenic activity against streptococcal agents (58).

Differences in probiotic strains, culture systems, and treatment procedures likely explain the variance in results obtained from tilapia fish study (58). The most effective tested formulation in this study for protecting tilapia against the highly pathogenic *S. agalactiae* was dietary *E. faecium* encapsulated with ALG-PG + 0.4% Fk. Because survival rates did not improve to 100%, more research into *E. faecium* in conjunction with other probiotic strains or various encapsulation matrixes may be worthwhile.

Dietary probiotics and more research in this field are anticipated to reduce the occurrence of dangerous bacteria in tilapia aquaculture, allowing for a more environmentally friendly growth of the tilapia breeding industry. Various analytical analyses of microencapsulated probiotic bacterial cells revealed great encapsulation efficiency and adequate survivability of probiotic cells in the simulated fish digestive system, as well as strong cell stability in all experimental gel formulations. Moreover, the finding of *in vivo* challenge test in the present report demonstrates that the microencapsulated *E. faecium* probiotic treatment with FOS and Fk could be used for treating *S. agalactiae* infected tilapia fish. This research represents a novel investigation to use a microencapsulated *E. faecium* probiotic-supplemented diet to control the mortality rate of *S. agalactiae* infected tilapia fish.

In conclusion, the probiotic *E. faecium* cells were successfully microencapsulated in appropriate sizes and shapes utilizing ALG-PG blends with varied concentrations of FOS and Fk. According to the findings, the ALG-PG + 0.4% Fk (F7) formulation had the highest encapsulation efficiency, viability in gastrointestinal conditions and during storage time, increased cell release, and excellent anti-pathogenicity against *S. agalactiae*. The survival rate of fish infected with S. agalactiae after 90 days of feeding with formulation F7 (0.6% ALG + 0.2% PG + 0.4% FK) was 63 ± 1.75%, while this amount for the un-encapsulated cell was 17 ± 1.25%. As shown in Table 4, the survival rate of fish was increased parallel to increasing of prebiotic percentage from 0.2 to 0.4%. Local herbal gums, such as PG and Fk, are indicated as a good scaffold and an appropriate matrix for probiotic encapsulation when combined with ALG. As a prebiotic, these herbal gums promote the growth of probiotic cells in the food environment and digestive tract. Meanwhile, the concentration of candidate probiotic in 33 samples of fish gastrointestinal tract and 33 samples of water in glass aquariums were 2.25 ± 0.73 × 10^7^ CFU g^−1^ and 3.56 ± 0.68 × 10^6^ CFU ml^−1^ respectively which proved the successful colonization of *E. faecium* ABRIINW.N7 in fish and the environment.

## Data availability statement

Publicly available datasets were analyzed in this study. This data can be found at: https://www.ncbi.nlm.nih.gov/nuccore/MK367697.

## Ethics statement

The animal study was reviewed and approved by Dr. Mahmood Reza Moradi (Chairman of the Academic/Regional Ethics Committee in Biomedical Research) and Dr. Farid Najafi (Secretary of the Academic/Regional Ethics Committee in Biomedical Research) of Kermanshah University of Medical Sciences (Approval ID: IR.KUMS.REC.1399.531).

## Author contributions

AK: designing experiment. YN and MK: writing. GL: data analysis. MJaf: revising. BH: project administrator. MJay and DE-A-K: providing revision. All authors listed have made a substantial, direct, and intellectual contribution to the lab work and approved it for publication.

## Funding

This work was supported by the Deputy for Research and Technology of Kermanshah University of Medical Sciences (Grant No. 990445).

## Conflict of interest

The authors declare that the research was conducted in the absence of any commercial or financial relationships that could be construed as a potential conflict of interest.

## Publisher's note

All claims expressed in this article are solely those of the authors and do not necessarily represent those of their affiliated organizations, or those of the publisher, the editors and the reviewers. Any product that may be evaluated in this article, or claim that may be made by its manufacturer, is not guaranteed or endorsed by the publisher.

## Abbreviations

FOS: fructooligosaccharide
S.A: *Streptococcus agalactiae*
PG: Persian gum
APS: Ammonium per sulfate
ALG: alginate
NN-MBAAm: *N*-methylene bis-acrylamide
BHIB: brain heart infusion broth
WG: weight gain
DWG: daily weight gain
RGR: relative growth rate
PWG: percent weight gain
SGR: specific growth rate.
